# Numerical simulation of deposition of drifts and salt from multiple super-large seawater cooling towers

**DOI:** 10.1038/s41598-023-36164-7

**Published:** 2023-06-22

**Authors:** Ziqi Liang, Lujun Li, Jie Yang, Qing Zhu

**Affiliations:** 1 State Key Laboratory of Nuclear Power Safety Monitoring Technology and Equipment, China Nuclear Power Engineering Co., Ltd., Shenzhen, Guangdong, China; 2 China Institute of Water Resources and Hydropower Research, Beijing, China

**Keywords:** Environmental sciences, Energy science and technology, Engineering

## Abstract

A three-dimensional CFD simulation model was established to study the characteristics of flow, drifts and salt deposition from 6 super-large seawater cooling towers in a power station. In the model, site meteorological data, design parameters of cooling tower, general layout, environmental characteristics, are considered. The results show that: (1) when the wind direction is parallel to the towers, the streams overlap, reducing deposition of drifts and salt onto the ground. (2) The drifts with particle size greater than 550 μm cannot float out of cooling towers. (3) In normal operation of 6 such cooling towers, the resulting salt deposition will not cause serious damage to plants.

## Introduction

With the rapid development of coastal industry and the increasing shortage of fresh water resources, the secondary circulation of large seawater cooling tower is often used as the main cooling method in coastal power plants^1^. The hot and humid air discharged from the seawater cooling tower contains small water droplets with large soluble salt content. They are carried out of the tower by the updraft to form drifts, which may cause corrosion on the surface of equipment pipes^2^, increasing the need of anticorrosion. It is also possible to cause the surrounding vegetation dryout and soil salinization, affecting the local ecology^3–6^. Such salt deposition issue prohibits the application and generalization of seawater cooling towers.

 In 1972, a series of studies were carried out in the United States, including cooling tower drifts, and numerical models such as ballistic model, Gaussian model and K theory model were proposed^7–9^. In 1990, the National Laboratory Center for Environmental Impact Research summarized previous models and field tests data to form the SACTI calculation program^10^.

The SACTI model was used to predict the plume dispersion discharging from cooling tower under normal operation and three different kinds of wind speeds by Guo RuiPing et. al.^11^. The results have shown that the plume length, plume height and plume radius are function of wind speed. The SACTI program still has drawbacks in simulating complex terrain. Therefore, when the buildings and terrain near the cooling tower are too complex, researchers tend to perform wind tunnel tests or field tests for a more realistic observation, notably of plume disperstion^12^.

In the recent decades, Computational Fluid Dynamics (CFD) has improved dramatically and also become a widely used method in the study of flow field around cooling tower and its pollutant dispersion considering complex buildings and terrain within the calculation domain. Its basic principle is to use discrete numerical method to solve the fluid physics. It has strong adaptability and is widely used in many engineering fields. The CFD method was used to study the influence of ambient wind on the exhaust steam of cooling tower by Takata^13^ and Meroney^14^, and the simulation results were compared with field measurement results, which proved good consistency between the two. Shi Xuefeng et. al.^15^ compared the CFD model and the SACTI model predictions of drifts and salt deposition in a cooling tower case, in which the CFD model considered evaporation, condensation, and local circulation and buildings, resulting in that the peak value of salt deposition predicted by the SACTI model was about 3 times as much as that of the CFD model. By comparing the simulation results of fog plume trajectory with the field observation results, CFD is deemed more reliable in flow field prediction, and suitable for the study of drifts and salt deposition problems.

In this study, CFD was applied to a group of six super-large seawater cooling towers in a power site to study the drifts characteristics in the cooling towers, the salt depostion and the resulting influence to the environment, considering realistic layout of the surrounding buildings and measured meteo data. The results provide technical reference for the selection of potential new sites, design of layouts, and for the assessment of the impact of cooling towers to the environment.

## The cooling towers

**Figure 1:**
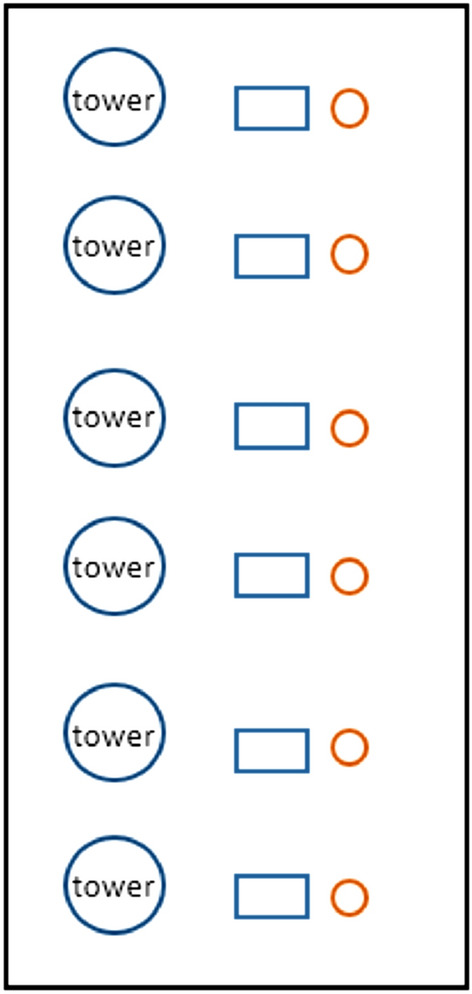
Arrangement of cooling tower group.

There are six cooling towers arranged in a north–south direction, with lower buildings on the east side of each cooling tower. The center spacing of the six towers from north to south is 250 m, 290 m, 250 m, 290 m and 250 m respectively, as shown in Fig. 1. The main dimensions of the cooling towers are shown in Table 1. "S-wave" fill of 2.0m height is used. The salinity of the circulating seawater is 28‰ and the concentration ratio is 1.5.

**Table 1:** Main size of cooling tower.

Parameter	Size	Parameter	Size (m)
Flood area	16000 m^2^	Outlet diameter	91.0
Tower height	211.9 m	Fill-top diameter	142.3
Inlet height	15.7 m	Fill-bottom diameter	143.2
Shell diameter	144.9 m	Fill-top elevation	21.06
Throat diameter	86.0 m	Strut diameter	155.8

## Numerical model

### Control function

The operating conditions including loads, meteo and the mass flowrate of the circulate water is assumed stable, the airflow field is assumed steady around the towers. The function of flow movement is shown in reference^16^. The porous media model of Fluent was adopted in the fill area, where the resistance loss coefficient of the fill is shown in Eq. (1). Discrete Phase Model (DPM) was used in rain zone, where the air resistance to raindrops was analyzed according to Eqs. (2) to (4). MERKEL model was used to analyze the heat exchange process between fill and rain zone.

1 $$\xi_{f} = \frac{{2A_{p} V^{M} \gamma_{a} }}{{\rho V^{2} H_{f} }}$$

2 $$\frac{{d\vec{u}_{p} }}{dt} = F_{D} \left( {\vec{V} - \vec{u}_{p} } \right) + \frac{{\vec{g}_{x} (\rho_{p} - \rho )}}{{\rho_{p} }}$$

3 $$F_{D} = \frac{18\mu }{{\rho_{p} d_{p}^{2} }}\frac{{C_{D} {\text{Re}} }}{24}$$

4 $$C_{D} = \frac{24}{{\text{Re}}}\left( {1 + b_{1} {\text{Re}}^{{b_{2} }} } \right) + \frac{{b_{3} {\text{Re}} }}{{b_{4} + {\text{Re}} }}$$

where *u*_*p*_ is particle velocity vector, m/s, *V* is air velocity vector, m/s, *g*_*x*_ is acceleration vector of particle, m/s^2^, *ρ*_*p*_ is density of raindrops, kg/m^3^, *d*_*p*_ is raindrop diameter, m, *C*_D_ is raindrop resistance coefficient, and *bi* is experience coefficient.

The commercial computational fluid software FLUENT was used to solve the three-dimensional numerical model of cooling tower. For natural-draft cooling tower, buoyancy is one of the main forces in momentum equation, so buoyancy effect was considered. Air flow, heat transfer, raindrop evaporation and water vapor diffusion need to be solved coupled. The discrete equation is solved by the separation of variables method, and the decoupling of velocity and pressure is performed by the SIMPLEC algorithm. QUICK format was adopted in discretization of velocity and temperature field. The second boundary conditions of outers, the resistance characteristics of the fill, and the evaporation of the raindrop are calculated by the secondary interface of FLUENT software, with user-defined function (UDF) enabled.

### Grid establishment

**Figure 2:**
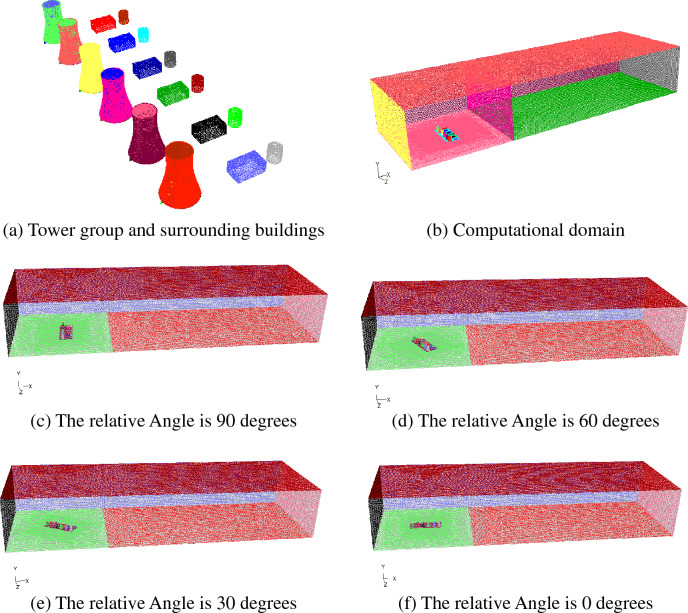
Grid in numerical model.

GAMBIT grid division software was used to establish a three-dimensional model, as shown in Fig. 2, with calculation range of 12500 m × 4500 m × 2500 m, divided into near field and far field, where the near field is divided into inside-tower and outside-tower, the outside-tower is divided into the perimeter and top, the inside-tower is divided into above-throat, below-throat, fill area, bottom of the tower and air inlet area. The grid of tower outside is sparse, and the fill area is the densest, with a total grid of about 8 million.

In order to calculate the trajectory of particles in different wind bearings, a set of grids were established with interval of 15° starting from 0° where the wind blows parrallely to the towers row, such that the distribution of salt deposition in all bearings and distances around the tower group could be conducted.

### Boundary condition

The computational domain is divided into two parts: inside and outside the cooling tower. For the boundary of the outer region, the bottom is an adiabatic boundary. When there is no wind, the other surfaces are pressure outlet boundaries. In case of ambient wind, the inlet surface is a velocity inlet boundary, and the other surfaces are pressure outlet boundaries. The cooling tower shell is arranged as an adiabatic boundary, and the inlet and outlet of the tower are arranged as the internal boundary. The fill is set to porous medium area, where the resistance coefficient is set according to the experiment experience. Meteorological parameters and operating parameters are shown in Table 2.

**Table 2:** Meteorological parameters and operating parameters.

Parameters	Winter	Spring and autumn	Summer
Dry-bulb temperature (°C)	0.68	13.08	23.58
Relative humidity	0.63	0.63	0.75
Atmospheric pressure (hPa)	1025.0	1017.2	1007.6
Circulating water volume (t/h)	134,568	134,568	134,568
Inlet water temperature (°C)	23.28	31.24	38.90

The inject surface of the raindrop is the filltop. The initial velocity, temperature and amount of raindrop are set in the DPM. which can be calculated by operating parameters. The motion characteristics of floating droplets under different particle size distribution conditions were studied. The drifts spectrum is shown in Table 3.

**Table 3:** The droplet spectrum.

Particle diameter(μm)	Mass fraction (%)	Particle diameter (μm)	Mass fraction (%)
30	18.75	450	0.86
50	18.10	500	0.76
70	10.13	600	1.27
90	7.84	700	1.07
110	6.74	800	0.80
130	5.92	900	0.68
150	5.37	1000	0.92
180	6.42	1100	0.19
210	4.98	1200	0.07
240	3.37	1300	0.05
270	2.18	1400	0.03
300	1.37	1500	0.02
350	1.42	1600	0.01
400	1.05	1700	0.01

In this study, the floating water rate of the water collector is 5 parts per million (5 × 10^–6^). The motion trajectory of a drift is calculated according to Eqs. (2) to (4). The evaporation of drifts is neglected when calculating the salt deposition.

**Figure 3:**
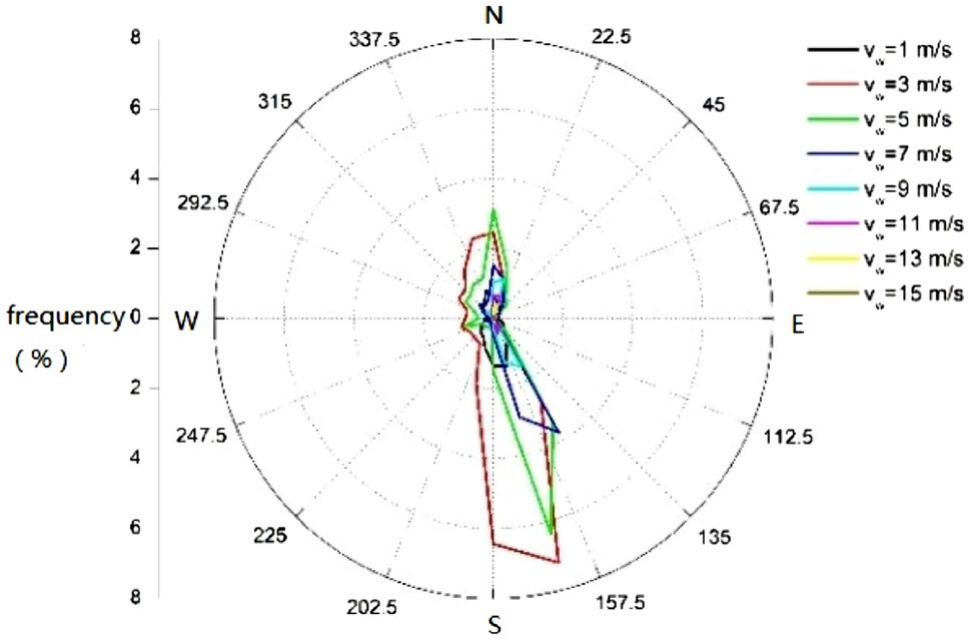
Wind rose of a whole year.

The annual or monthly distribution of salt deposition is closely related to the distribution of deposited quantity of drifts at each wind direction and wind speed Fig. 3 shows the annual wind rose diagram, in which 1 m/s represents the wind speed interval of 0 ~ 1 m/s, 2 m/s represents the wind speed interval of 1 ~ 2 m/s, etc. The ambient wind speed is correlated with the height, as shown in Eq. (5), the wind shear index is related to site and atmospheric stability category, here for example in neutral category is 0.19^17^. The UDF function is used to setup the wind speed vertical profile. To characterize the influence of ambient humidity, air moisture content and temperature were set in the pressure outlet boundary or velocity inlet boundary.

5 $$v_{w,y} = v_{w} \left( {\frac{y}{{h_{ref}^{{}} }}} \right)^{0.19}$$

where *v*_*w,y*_ is wind speed measured *y* meters above the ground, m/s, *v*_*w*_ is wind speed measured at reference height, m/s, *h*_*ref*_ is the reference height, 10 m.

### Data statistical method

For each grid, regular lattices of 50 m × 50 m  on ground-level are set-up to count the quantity of deposited drifts, such that for each wind direction, each wind speed and each released drifts size, the total deposited mass of drifts are calculated, and afterward the deposition of salt in each bearing and distance can be conducted, as Eq. (6).

6 $${\text{q}}_{{\text{i}}} { = }\sum\limits_{j = 1}^{j = N} {\frac{{{\text{m}}_{{{\text{d}}_{j} }} }}{{50 \times 50 \times \rho_{{{\text{d}}_{j} }} }} \cdot 3600 \cdot 1000 \cdot {\text{f}}_{{{\text{d}}_{j} }} \cdot {\text{f}}_{{{\text{wind}}}} }$$

where *q*_*i*_ is the amount of droplets in the *i* region, mm/h; *m*_*dj*_ is the mass of droplets with particle diameter of *d*_*j*_ falling in this region, kg/s; ρ_*dj*_ is the density of droplets with particle diameter of *d*_*j*_, kg/m^3^; *f*_*dj*_ is the frequency of droplets with particle diameter of *d*_*j*_, kg/kg; *f*_*wind*_ is the wind frequency of the corresponding direction in the region.

## Result analysis

### Flow field

**Figure 4:**
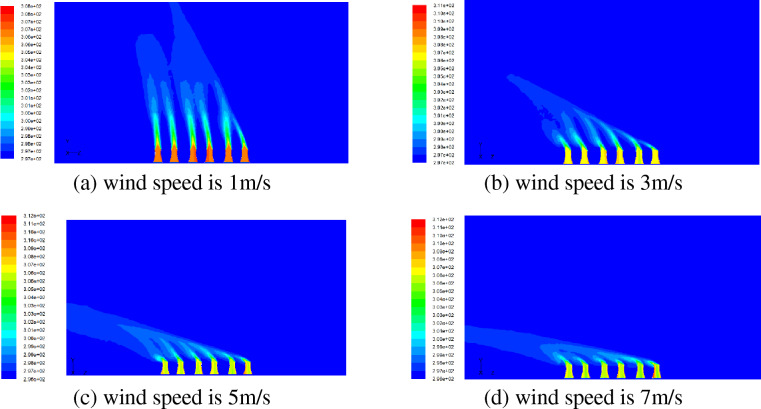
Flow field when the wind direction is parallel to tower row (K, wind direction 0°).

Figure 4 shows the flow characteristics of inside and outside of cooling towers when the ambient wind direction is parallel to the tower row. When the wind speed is 1 m/s, the hot air from the first cooling tower outlet on the windward side is obviously deflected, while the hot air from other cooling towers outlets is not obviously deflected. When the wind speed is 3 m/s, the outlet air deflection of the first and second cooling towers on the windward side is large, while the outlet air deflection of other cooling towers is relatively small. When the wind speed is greater than 5 m/s, the air from the cooling tower outlets has a large deflection, while the leeward side is slightly smaller. When the wind direction is parallel to the tower row, the cooling tower on the windward side can play a protection role and reduce the deflection range of hot air from the leeward side cooling tower outlet.

**Figure 5:**
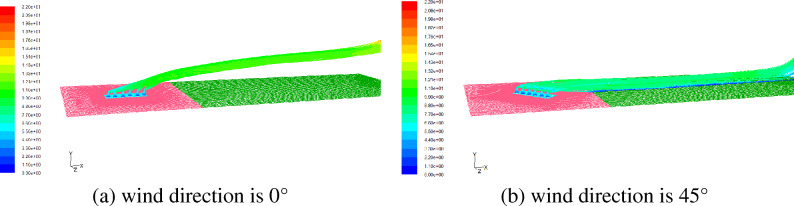
Pathline from tower outlet when the wind speed is 5 m/s.

Figure 5 shows the pathline of hot and humid air from cooling tower outlet under the wind speed of 5 m/s with different wind directions. As can be seen from the velocity distribution of outlet air flow in Fig. 5, when the wind direction is parallel to the tower row, the overlapping of hot air at the outlet leads to the larger velocity of drifts. From the trajectories, it can be seen that when the wind direction is parallel to the tower row, are supported by the air flow to move in a higher region. When the wind direction is 45 degrees away from the tower row, the air flow is not superimposed, at this time, the drifts is obviously slower, and are more likely to fall, resulting in salt deposition. When the wind direction is at some certain angle, the hot air from outlet of the six cooling towers does not overlap, and strongly deflects under the act of wind.

### Drifts trajectory with different particle sizes

**Figure 6:**
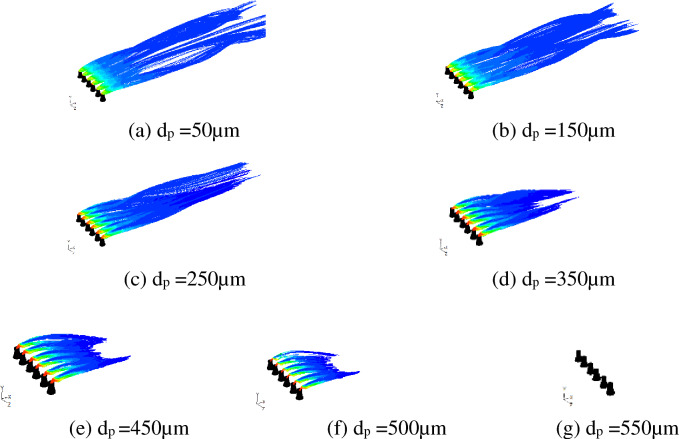
Droplet trajectories of different particle diameter at cooling tower openings.

Figure 6 shows the escape tracks of drifts with different particle sizes. When the partical diameter is small, the drifts barely drop. When the particle diameter is about 350 μm or larger, the escaped drifts drop to the ground within an obviously shorter distance. When the particle diameter is 550 μm or larger, drifts cannot escape from the cooling tower outlet.

**Figure 7:**
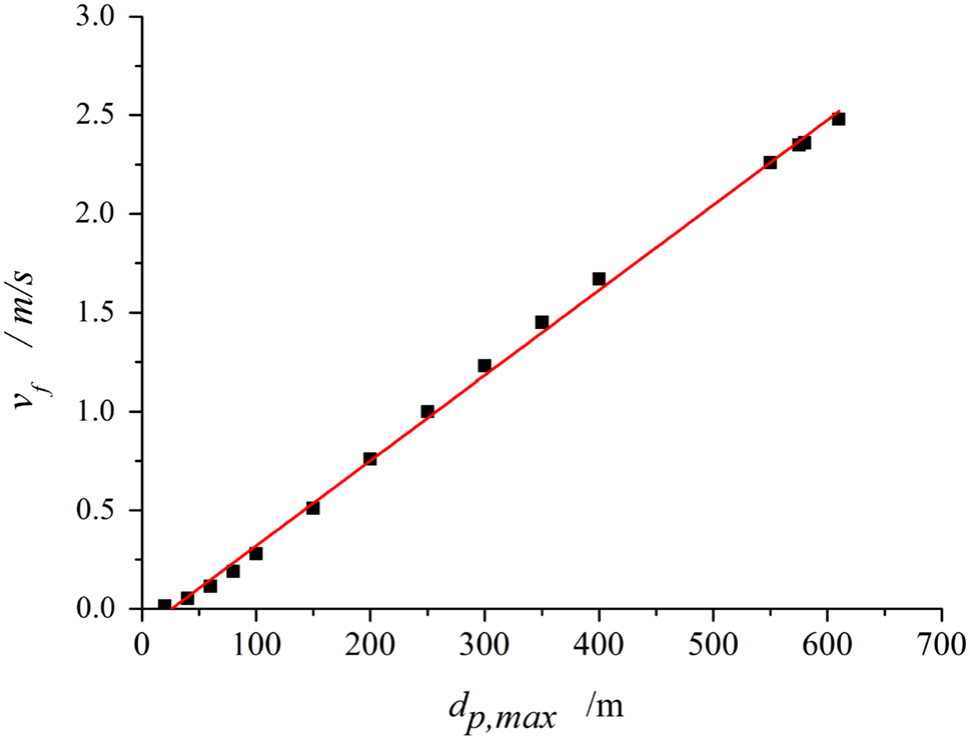
Relationship between the maximum diameter of floating droplets and the wind speed of the fill section.

The interaction between air and raindrop is related to raindrop diameter and relative velocity. If the air velocity inside the tower is low or the diameter of the raindrops is large, the raindrops will not be able to float out of the tower. Theoretically, only when Eq. (2) is greater than 0, raindrops are able to float out of the tower. Therefore, the relationship between the maximum particle diameter of floating droplets and the wind speed of the fill section is shown in Fig. 7. When the wind speed of the fill section is 2 m/s, the maximum particle diameter allowing drifts to float out is about 500 μm, which is in good agreement with the above numerical analysis. It is proved that the numerical model can predict the motion trajectory of floating droplets well.

### Distribution of salt deposition

**Figure 8:**
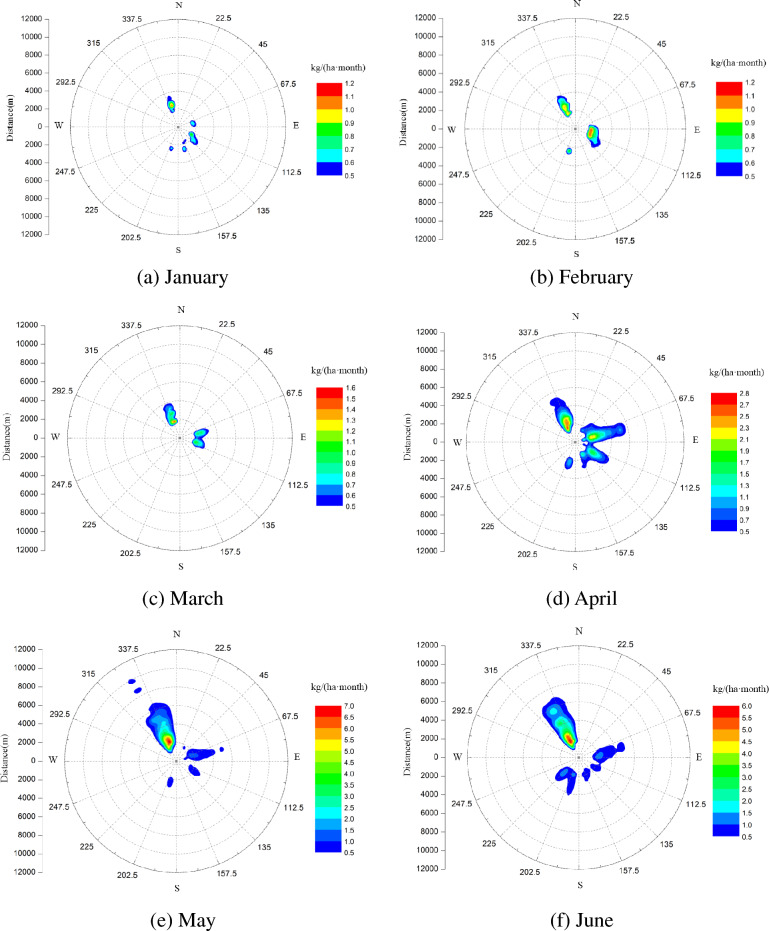

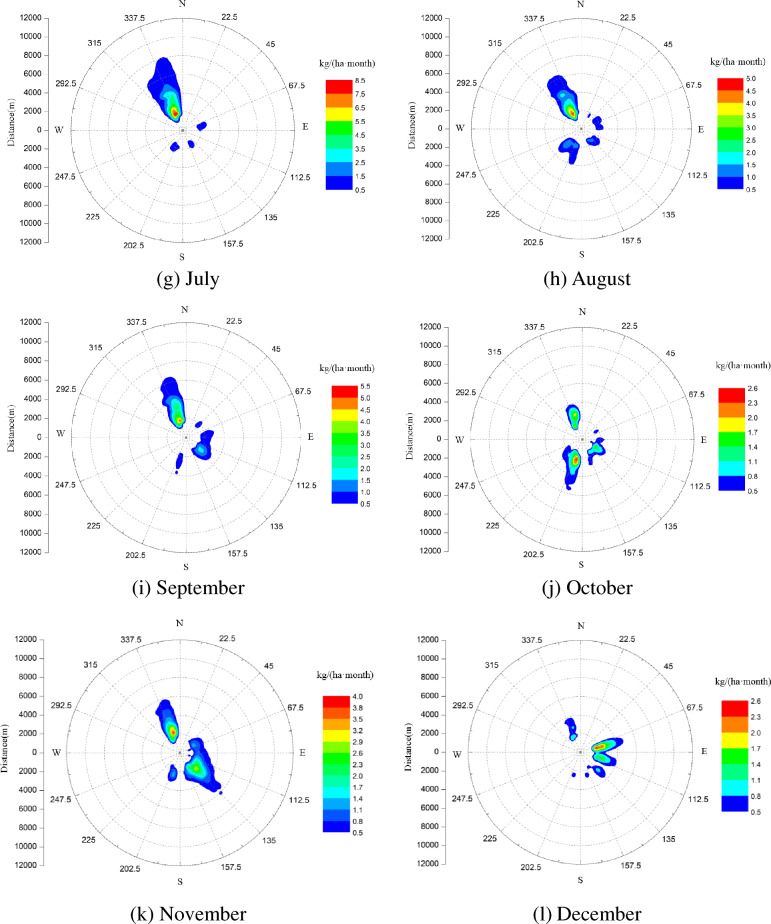
Seasonal cumulative distribution of salt deposits.

According to a NUREG report, salt deposition of 10 kg/(ha month) or more in any single month of plant-growing seasons may lead to leaf damage of many species. Figure 8 shows the predicted distribution of salt deposition from the group of six simultaneously-operating super-large seawater cooling towers, the deposits mass is accumulated to each month. It can be seen that the highest peak of salt deposition happens in July and is about 8.5 kg/(ha month), located at NNW and about 2 km away from the site. The peak of salt deposition in January and February is the smallest, about 1.2 kg/ (ha month), located around NNW and ENE, 2 km away from the site. It can be seen that the peak of salt deposition is generally larger in summer (June, July and August), followed by spring (March, April and May), and smaller in winter (December, January and February).

**Figure 9:**
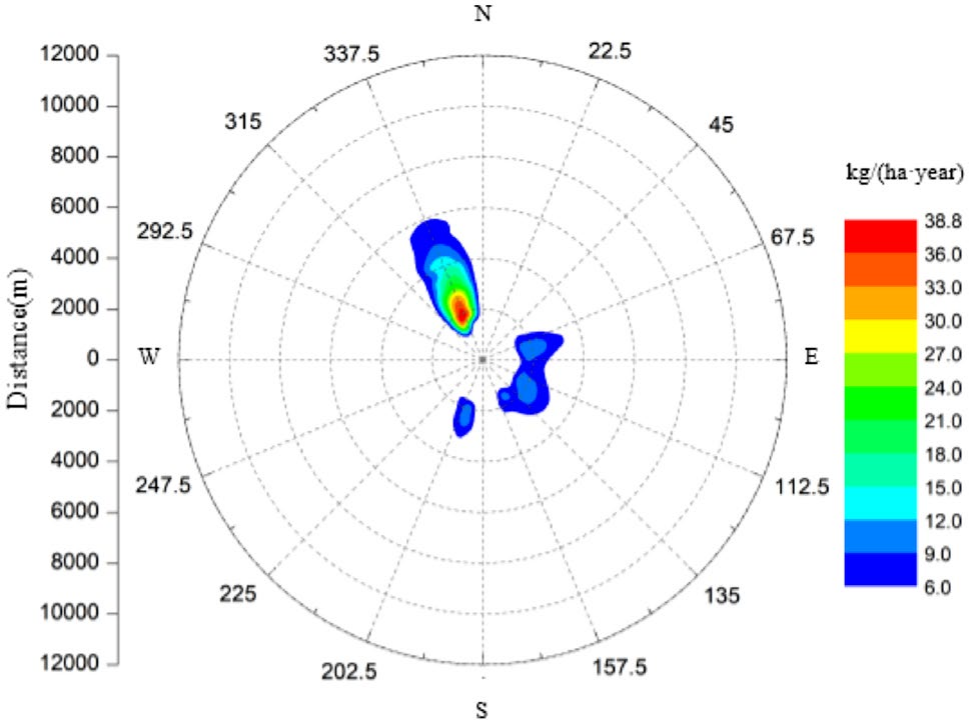
Annual cumulative distribution of salt deposits.

When salt deposition exceeds 200 kg/(ha·year), it is considered that it will cause serious damage to plants, and preventive measures should be taken. Figure 9 shows the predicted distribution of salt deposits during the year. The annual peak salt deposition is about 38.8 kg/(ha·year), which occurs at NNW, about 2–3 km away from the site.

## Conclusion

A three-dimensional numerical simulation with CFD method was adopted to analyze the air flow and drifts trajectories from a group of six super-large seawater cooling towers. Distribution of drifts and salt depostion was couducted, and the impact of salt depostion to the environment was assessed. Main conclusions are as follows:

1. When the wind direction is parallel to the tower row, the cooling tower on the windward side can play a shielding role, reducing the deflection amplitude of the hot air at the outlet of the cooling tower on the leeward side. The hot air at the outlet overlap to form as one airflow, which enhances dispersion, therefore flattening the deposition peak of drifts and salt.
2. When the particle diameter of drifts is greater than 550 μm, they cannot float out of the cooling tower.
3. Under normal operation conditions of the 6 super-large seawater cooling towers, the salt deposition reaches its maximum in summer, while minimum in winter. The monthly peak of salt deposition is predicted less than 8.5kg/(ha month), which would not cause damages to leaves. The annual peak of salt deposition is predicted 38.8 kg/(ha·year) less than 200 kg/(ha·year), which would not cause serious damage to plants.

## Data Availability

The data used to support the findings of this study are available from the corresponding author upon request.

## List of symbols

*u*_*p*_: Particle velocity vector, m/s
*V*: Air velocity vector, m/s
*g*_*x*_: Acceleration vector of particle, m/s^2^
*ρ*_*p*_: Density of raindrops, kg/m^3^
*d*_*p*_: Raindrop diameter, m
*C*_D_: Raindrop resistance coefficient
*bi*: Experience coefficient
*v*_*w,y*_: Wind speed measured at *y* metres above the ground, m/s
*v*_*w*_: Wind speed measured at reference height, m/s
*h*_*ref*_: Reference height, m, 10m is used
*y*: Height, m
